# Classifying post-traumatic stress disorder using the magnetoencephalographic connectome and machine learning

**DOI:** 10.1038/s41598-020-62713-5

**Published:** 2020-04-03

**Authors:** Jing Zhang, J. Don Richardson, Benjamin T. Dunkley

**Affiliations:** 1 Department of Diagnostic Imaging, Hospital for Sick Children, Toronto, ON Canada; 2 Neurosciences & Mental Health, SickKids Research Institute, Toronto, ON Canada; 3 St Joseph’s, London OSI, London, ON Canada; 4 MacDonald Franklin OSI Research Centre, London, ON Canada; 5 Department of Medical Imaging, University of Toronto, Toronto, ON Canada

**Keywords:** Biomarkers, Translational research

## Abstract

Given the subjective nature of conventional diagnostic methods for post-traumatic stress disorder (PTSD), an objectively measurable biomarker is highly desirable; especially to clinicians and researchers. Macroscopic neural circuits measured using magnetoencephalography (MEG) has previously been shown to be indicative of the PTSD phenotype and severity. In the present study, we employed a machine learning-based classification framework using MEG neural synchrony to distinguish combat-related PTSD from trauma-exposed controls. Support vector machine (SVM) was used as the core classification algorithm. A recursive random forest feature selection step was directly incorporated in the nested SVM cross validation process (CV-SVM-rRF-FS) for identifying the most important features for PTSD classification. For the five frequency bands tested, the CV-SVM-rRF-FS analysis selected the minimum numbers of edges per frequency that could serve as a PTSD signature and be used as the basis for SVM modelling. Many of the selected edges have been reported previously to be core in PTSD pathophysiology, with frequency-specific patterns also observed. Furthermore, the independent partial least squares discriminant analysis suggested low bias in the machine learning process. The final SVM models built with selected features showed excellent PTSD classification performance (area-under-curve value up to 0.9). Testament to its robustness when distinguishing individuals from a heavily traumatised control group, these developments for a classification model for PTSD also provide a comprehensive machine learning-based computational framework for classifying other mental health challenges using MEG connectome profiles.

## Introduction

Armed Forces members, due to the nature of their work, represent an at-risk group to develop posttraumatic stress disorder (PTSD). PTSD is a chronic psychiatric condition which can occur after being exposed to a potentially traumatic event including exposure to actual or threatened death, serious injury or sexual violence, learning that this (event) occurred to a close relative or close friend, or experiencing repeated or extreme exposure to aversive details of the event^1,2^. The consequences to PTSD include prolonged suffering, distress, impaired quality of life and increased mortality^3^. The disorder is a major neuropsychiatric disorder among military personnel, with up to 17% of Canadian Armed Forces members developing PTSD within the first-year post-deployment^4^. The current gold standard for PTSD diagnosis is based on Diagnostic and Statistical Manual of Mental Disorders (updated version: fifth edition, or DSM-V^1^). Along with DSM-IV^5^ used for the subjects in the current study, these protocols rely heavily on the subjective report of the patients and, given the stigma of a diagnosis in some groups, or difficulty articulating their symptoms, a clear diagnosis can be difficult. As such, an objective diagnosis platform is highly desirable.

One critical step of developing such a framework for PTSD is understanding its psychophysiological and molecular pathology. The underlying neurobiological pathogenesis is increasingly understood within the context of dysfunctional brain circuits^6^. A mechanism that mediates communication and information processing within and between brain circuits is neural oscillations and synchrony^7^. Magnetoencephalography (MEG) can image these phenomena non-invasively, and has been used as an effective research tool for exploring the neural activity associated with various neurodegenerative and neuropsychological disorders, including depression, bipolar disorder, mild traumatic brain injury (mTBI) and Alzheimer’s disease^8–11^ as well as PTSD-related functional circuitry^12–15^. At the group level, neural synchrony can stratify those with PTSD from a heavily traumatised, but otherwise matched, control group^15^, with hippocampal synchrony directly related to symptom severity across individuals^14^. This suggests synchrony might be a reliable signature for PTSD identification.

Rapid advancement in artificial intelligence and machine learning have shown promise in brain imaging and computational neuroscience. Various Bayesian inference-based machine learning algorithms have been developed and implemented for neuroimaging signal processing and temporal brain activity prediction^16^. In translational research and clinical applications, these methods are being actively explored for pre-symptomatic diagnosis, prognostic prediction, and medical intervention effectiveness prediction^17^. Neurodegenerative and neuropsychological disorders like Huntington’s disease, mTBI and bipolar disorder are among the examples with promising results^17–19^.

The objective here was to implement a machine learning classification modelling workflow for delineating individuals with PTSD from trauma-exposed, matched control participants using MEG-derived functional connectomes based on neural synchrony. We developed a comprehensive machine learning pipeline based on support vector machine (SVM) and random forest (RF) algorithms, leveraging their classification modelling and feature selection capabilities, respectively. We recruited combat-related PTSD and the same combat trauma-exposed control participants from the Canadian Armed Forces, data that has been published in previous studies^14,15^. This design builds upon our established work and also takes advantage of the similar contexts of traumatic exposure and chronic stress present across participants from serving in a military context, as compared with those from a civilian setting. The present study tests the utility of machine learning in differentiating PTSD and traumatised control groups in the context of military-combat specific cases.

## Results

**Figure 1:**
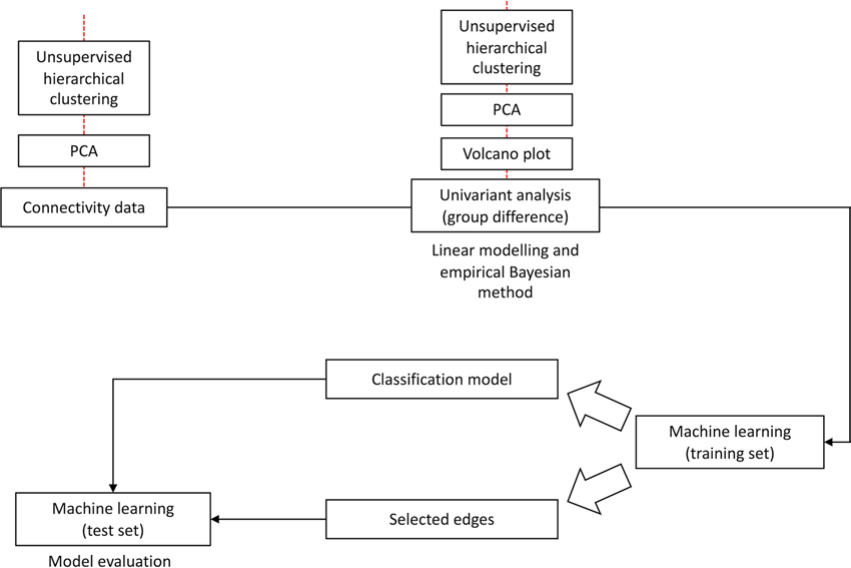
Flowchart for the overall process of machine learning MEG synchrony discovery framework.

**Figure 2:**
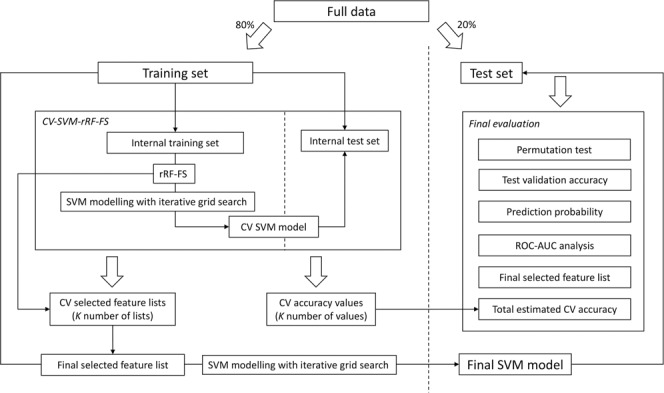
Flowchart for the workflow of the SVM modelling analysis.

Following the workflow described in Figs. 1 and 2 and Supplementary Information S1, below are the results from the key steps of the downstream analysis process.

### Univariate analysis

**Figure 3:**
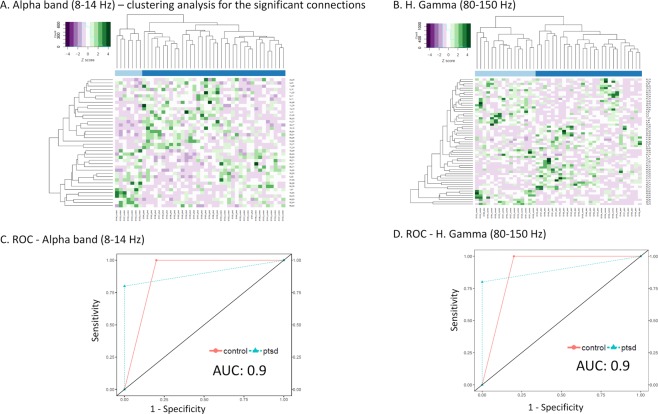
Heatmaps for hierarchical clustering analysis results using only the significant edges (p < 0.01) as well as ROC-AUC results for the Alpha and H. Gamma bands. For heatmaps, dendrograms show the clusters for participants (columns) and the edges (rows), and z score was plotted. For ROC, AUC values are shown on the plot. (**A**) Alpha band heatmap, (**B**) H. Gamma band heatmap, (**C**) Alpha band ROC, and (**D**) H. Gamma band ROC.

A summary of the univariate analysis results can be viewed in Table 1. The complete univariate analysis along with clustering analysis results are included in Supplementary Information S2 and Table S1. Hierarchical clustering was conducted on the data with only the significant edges (p < 0.01) to assess the clustering pattern upon univariate analysis (Figs. 3A,B and S3). In general, data with only the significant edges showed improved group clustering results for the five frequency bands. However, we also observed varying results corresponding to specific frequency bands. Regarding the Alpha band (Fig. 3A), the clustering result grouped seven participants from the control group into a major cluster, with the second major cluster containing the rest. Within the second major cluster, most PTSD (post-traumatic stress disorder) participants were grouped together. For H. Gamma (high gamma) band, with an improved grouping pattern (Fig. 3B), the control group exhibited a higher-level of variance than the PTSD group, where six control participants where included in the PTSD cluster. Moreover, the Theta band data managed to mostly separate the PTSD participants from control **(**Fig. S3A). As seen in Fig. S3B, the Beta band also showed substantially improved PTSD and control group clustering with only the significant edges where only two PTSD participants were placed in the control group. For L. Gamma band (Fig. S3C), although two clusters were identified mostly according to the participant groups, the first major cluster only included five control participants, with the rest clustered with the PTSD group to form the second major cluster.

**Table 1:** Univariate analysis and CV-SVM-rRF-FS results summary.

Frequency band	Significant edges (p < 0.01)	Increase	Decrease	CV-SVM-rRF-FS selected edges	Increase	Decrease
Theta	30	15	15	11	1	10
Alpha	40	22	18	14	9	5
Beta	49	30	19	20	10	10
L. Gamma	22	7	15	12	4	8
H. Gamma	59	26	33	19	9	10

### Machine learning analysis

The final SVM (support vector machine) models were evaluated by permutation test, 10-fold CV (cross validation), as well as the external test set (from the initial random data partitioning step) (Figs. 3C,D, S4 and S5). The permutation test suggested that the prediction accuracy of the original model was higher than all the permutation models, leading to a permutation p value at 0.01 across the five frequency bands tested (Fig. S5). A summary of the CV-SVM-rRF-FS (cross validation with support vector machine and recurrent random forest feature selection) results can be viewed in Table 1; and the full list of CV-SVM-rRF-FS selected edges can be viewed in Table 2. A complete description for SVM modelling results can be found in Supplementary Information S2 and Table S2, including performance of ten CV models (mean classification accuracy and standard deviation, SD).

**Table 2:** Edges selected by the SVM/rRF-FS process. Abbreviations: inf, inferior; sup, superior; mid, middle; orb, orbital; tri, pars triangularis; oper, operculum; ant, anterior.

Frequency band	Region 1	Region 2
Theta	Lingual.L	Temporal.Sup.R
	Frontal.Mid.R	Insula.L
	Frontal.Mid.R	Postcentral.R
	Supp.Motor.Area.L	Occipital.Mid.L
	Frontal.Inf.Tri.R	Temporal.Mid.R
	Fusiform.L	SupraMarginal.R
	Frontal.Inf.Oper.R	Thalamus.L
	Frontal.Inf.Oper.R	Lingual.L
	Frontal.Inf.Tri.R	Rectus.R
	ParaHippocampal.R	Parietal.Inf.L
	Frontal.Mid.L	Temporal.Inf.R
Alpha	Parietal.Sup.R	SupraMarginal.R
	Temporal.Mid.L	Temporal.Pole.Mid.L
	Precentral.L	Frontal.Inf.Tri.L
	Frontal.Sup.Medial.R	Temporal.Pole.Mid.R
	Lingual.R	Caudate.L
	Temporal.Pole.Sup.L	Temporal.Mid.L
	Frontal.Inf.Oper.L	Olfactory.R
	Angular.R	Caudate.L
	Frontal.Inf.Oper.L	Thalamus.R
	Olfactory.R	Calcarine.L
	Frontal.Sup.Medial.L	Cuneus.L
	SupraMarginal.R	Heschl.R
	Temporal.Pole.Mid.R	Putamen.R
Beta	Precentral.L	Frontal.Mid.Orb.L
	Frontal.Inf.Orb.L	Temporal.Inf.L
	Rectus.L	Temporal.Inf.L
	Amygdala.L	Fusiform.L
	Lingual.R	Thalamus.R
	Parietal.Sup.L	Precuneus.R
	Rolandic.Oper.L	Temporal.Pole.Sup.L
	Rolandic.Oper.L	Putamen.L
	Rolandic.Oper.R	Occipital.Sup.R
	Cingulum.Ant.R	Pallidum.R
	Rolandic.Oper.L	Insula.L
	Cingulum.Mid.R	SupraMarginal.L
	Frontal.Sup.R	Frontal.Sup.Orb.R
	Frontal.Inf.Tri.R	Temporal.Mid.R
	Frontal.Sup.Medial.L	Postcentral.R
	Frontal.Sup.R	Paracentral.Lobule.L
	Frontal.Mid.L	Fusiform.R
	Hippocampus.R	Temporal.Pole.Mid.L
	Fusiform.L	Putamen.R
L. Gamma	Cingulum.Mid.L	Caudate.R
	Hippocampus.L	Temporal.Pole.Mid.R
	Frontal.Mid.Orb.R	SupraMarginal.L
	ParaHippocampal.R	Angular.L
	Occipital.Inf.R	Angular.L
	Frontal.Inf.Tri.L	Heschl.R
	Rectus.R	Occipital.Inf.L
	Frontal.Mid.Orb.L	ParaHippocampal.L
	Frontal.Mid.Orb.L	Paracentral.Lobule.L
	Lingual.L	Temporal.Inf.R
	Olfactory.L	SupraMarginal.L
	Calcarine.L	Parietal.Inf.R
H. Gamma	Insula.R	Parietal.Inf.R
	Parietal.Inf.L	Pallidum.R
	Cingulum.Post.R	Hippocampus.L
	Parietal.Inf.L	Temporal.Inf.L
	Cingulum.Mid.L	Parietal.Inf.R
	Cingulum.Post.R	ParaHippocampal.L
	ParaHippocampal.R	Temporal.Pole.Sup.R
	Temporal.Mid.R	Temporal.Pole.Mid.R
	Occipital.Mid.R	Temporal.Pole.Mid.L
	Frontal.Mid.Orb.L	Parietal.Sup.L
	Rolandic.Oper.R	Parietal.Inf.R
	Frontal.Inf.Orb.L	Amygdala.L
	Supp.Motor.Area.R	Temporal.Mid.R
	SupraMarginal.L	Thalamus.R
	Frontal.Inf.Oper.L	Cuneus.L
	Frontal.Inf.Oper.R	Paracentral.Lobule.R
	Frontal.Sup.R	ParaHippocampal.R
	Precuneus.R	Heschl.L

Here we use Alpha and H. Gamma as examples to exhibit the FS and SVM modelling results. For Alpha, the rRF-FS step nested CV process identified 14 edges as the most relevant features for PTSD-control stratification, including edges between the right superior parietal lobe and supramarginal gyrus, left middle temporal gyrus and middle temporal pole, as well as left precentral gyrus and inferior frontal gyrus pars triangularis. The final Alpha band SVM model was then trained with 31 support vectors with an internal CV accuracy of 0.94 ± 0.12 (mean ± SD). Upon evaluating with external testing data, the AUC value for the Alpha band was 0.9 (Fig. 3C). For H. Gamma, 19 edges were selected by the rRF-FS step as the most important features, including edges involving the left amygdala, left hippocampus and thalamus. The nested CV also led to an internal CV accuracy at 0.94 ± 0.11. The AUC value was determined at 0.9 for H. Gamma band (Fig. 3D).

### Principal component analysis

**Figure 4:**
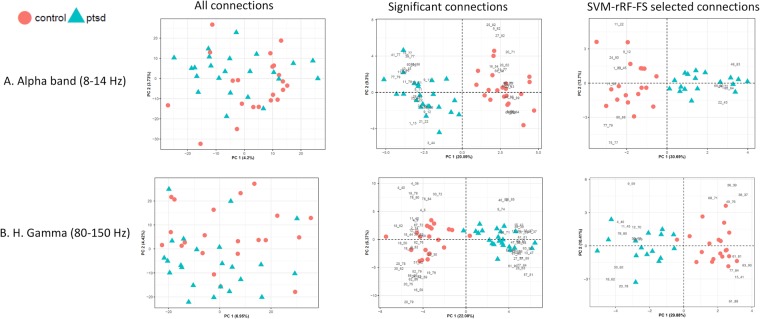
Score plots and biplots (i.e. score plot and loading plot) showing PCA result. For biplot, the loading plots exhibit the contribution of the edges to clustering pattern. Left column: Score plots for PCA results from all the edges; middle column: biplots for PCA results from feature-reduced data with only the significant edges (p < 0.01); right column: biplots for PCA results from data with CV-SVM-rRF-FS selected edges. (**A**) Alpha band and (**B**) H. Gamma band.

Serving as an unsupervised clustering and data complexity assessment tool, PCA was conducted at various points of the downstream data analysis for the five frequency bands tested. The results can be viewed in Figs. 4 and S6. For all five frequency bands, when using all edges, PCA failed to separate the PTSD group from the control participants, whereas the significant edges and feature selected datasets showed substantially improved group clustering. Due to the reduced data dimensionality for the feature-reduced data sets, the data complexity also decreased considerably, which is demonstrated by the increase of the percentage variance explained by the first two PCs in the PCA results.

### Partial least squares discriminant analysis

**Figure 5:**
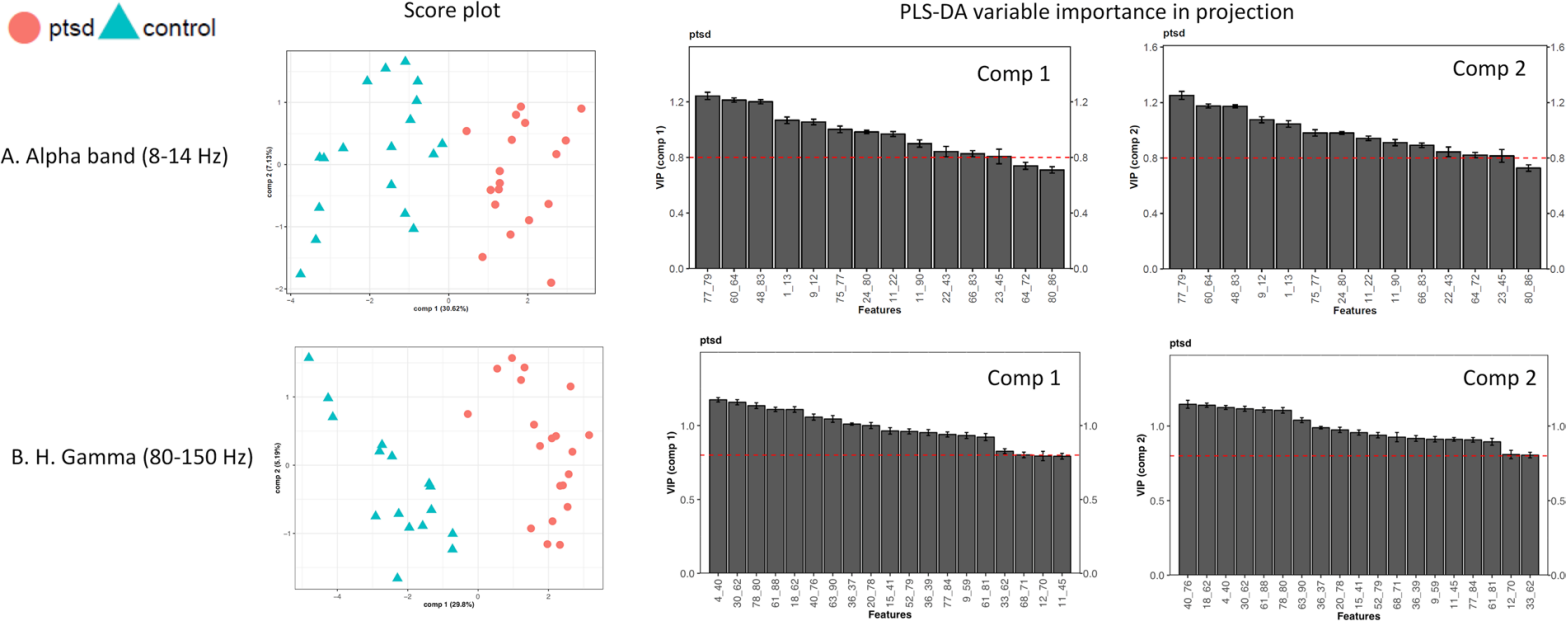
Score plots and VIP plots showing PLS-DA results. Left column: PLS-DA score plots showing the supervised clustering pattern on both components upon PLS-DA modelling using the CV-SVM-rRF-FS selected edges; right column: PLS-DA VIP scores for both model components for all the CV-SVM-rRF-FS selected edges, with the horizontal dashed line indicating the importance threshold (0.8), and the codes on the x-axis representing the edges. (**A**) Alpha band and (**B**) H. Gamma band.

PLS-DA results can be viewed in Supplementary Information S2, as well as Figs. 5, S7 and S8.

## Discussion

Due to the subjective nature of PTSD (post-traumatic stress disorder) diagnostics, the overlap of PTSD symptoms with other disorders (concussion, for example^20^), and the high comorbidity of other diseased states, including anxiety and depression^21^, an objectively measurable signature for diagnosing PTSD is desirable. Our research has important clinical implications as this type of platform, in concert with the conventional interview and questionnaire-based PTSD diagnostic methods, increases the diagnostic accuracy and facilitate personalized medicine. PTSD shares common symptoms with major depressive disorder as well as mild traumatic brain injury and improving the diagnostic accuracy will help decrease the heterogeneity of PTSD and thereby improving our understanding of the neurobiology of PTSD. Given the complexity and dynamic repertoire of neural activity, additional qualifications for an optimal fingerprint include being non-invasive, neurobiologically-informed, as well as high-throughput – this can be achieved by leveraging functional neuroimaging in combination with machine learning and artificial intelligence-inspired approaches.

We considered macroscopic neural circuits based on MEG (magnetoencephalographic) synchrony as one fingerprint source. Indeed, MEG has been used to understand human neurophysiology, including a wide range of neurodegenerative and neuropsychological disorders, such as mTBI, Alzheimer’s disease, depression and bipolar disorder^9–11^. Neural oscillations recovered using MEG are known to be dysfunctional in PTSD and correlate with primary symptoms and secondary complaints in the disorder^12–15,22^. Building on this, we explored the utility of a feature selection (FS) and machine learning-based classification modelling pipeline on PTSD biomarker discovery and classification. Specifically, we conducted machine learning analysis on a PTSD/traumatized control dataset with over 4000 unique functional edges, across a number of neurophysiological frequency ranges that are used for multiplexed communication in the brain. With a traumatized control group that had experienced similar combat-related stress as the PTSD cohort, our study speaks to the robustness of our machine learning classification framework.

Unsupervised cluster analysis and univariate statistical analysis were used to assess the overall property of the data and the group difference. Hierarchical clustering analysis conducted on all the edges showed that the complete functional profiles failed to exhibit any grouping patterns across any of the frequency bands. Moreover, the PCA (principal component analysis) results were found to be in complete agreement with the hierarchical clustering analysis, where the score plot exhibited substantial level of overlap among participants from the two groups. These results are at least consistent with the similar life experience the participants with PTSD and control participants had in their military training, deployment and experience of chronic stress and acute trauma during frontline deployment - it is not surprising that the groups possess similar superficial functional profiles in neural activity indexed by synchrony. The univariate analysis identified the significant data features for all the frequency bands, with and average number of remaining features at 40 ± 15 (mean ± SD), a hundred-fold reduction from the original 4005 edges. The number of significant edges (p < 0.01) fluctuated according to frequency band, showing frequency-specific patterns. For example, L. Gamma band exhibited the least amount of significant edges (p < 0.01), suggesting that the PTSD functional profiles might contain more individual variance across the two participant groups. H. Gamma (high gamma) included the largest number of significant edges (p < 0.01), consistent with our previous findings where substantial group difference in neural synchrony was identified for the H. Gamma rhythm^14,15^.

With only significant edges, the hierarchical clustering and PCA results showed drastically improved group clustering patterns. Specifically, hierarchical clustering exhibited almost complete clustering for the two groups in Theta, Beta and L. Gamma (low gamma). Even though the Alpha and H. Gamma bands failed to exhibit similar results, the clustering analysis still saw a clear trend of grouping the participants by diagnostic label. Despite the similarity of the complete functional profiles when comparing the two groups, our parametric univariate analysis workflow was able to capture the subtle differences between the two groups. Furthermore, the PCA results suggested that the two groups could be mostly separated on the first PC. Naturally, the data complexity was also greatly reduced for the data with less features. Consistent with the hierarchical clustering results, PCA also exhibited frequency-specific patterns. Moreover, PCA showed the groups were separated on PC1, which explained the most percentage data variance (around 20%), suggesting the patient/control grouping was the most crucial variable differentiating the data.

The machine learning analysis was conducted for all five frequency bands. Only using the training data, the CV-SVM-rRF-FS (cross validation with support vector machine and recurrent random forest feature selection) step substantially reduced the number of edges across all five frequency bands (Table 1). Despite such reduction in data dimensionality, clustering performance stayed mostly unchanged from the data with only the significant features, as seen in the PCA results. This indicates that the current machine learning feature selection strategy was capable of effectively reducing the dimensionality of the data while preserving the information necessary to separate PTSD participants from the control group. Moreover, the PLS-DA VIP (partial least squares discriminate analysis variable importance in projection) evaluation independently confirmed the importance of the selected features. These suggests that our feature selection process was subject to minimal bias.

Frequency-specific patterns were observed for the feature selection results. First, the quantity of the selected edges followed a similar trend as the univariate analysis results, where Beta and H. Gamma exhibited the most selected features, whereas the Theta and L. Gamma bands showed the least. Moreover, based on the corresponding univariate analysis results, we evaluated the directionality distribution for the CV-SVM-rRF-FS selected edges. For example, Theta showed decreased synchrony for 10 out of 11 total selected edges when comparing the PTSD group with controls. Interestingly, a previous study of ours revealed an increase in synchrony in PTSD when compared to controls under the same frequency band, but in a task-dependent manner, during a cognitive flexibility protocol^23^. This suggests the repertoire of neurophysiological activity in PTSD in this particular frequency band is highly dynamic and flexibly modulated by task. Whilst not examined here, it also suggests using task-induced changes in neural synchrony might be ripe for use as features in machine learning classification for mental illness, as this highly dynamic neural activity is essentially untapped in resting state paradigms. In any case, the initial univariate analysis here revealed an equal number of edges with significant increases (p < 0.01) and decreases in synchrony, whereas, in addition to drastically reducing the number of edges, CV-SVM-rRF-FS selected more edges with decreased connectivity. These confirmed that while the univariate analysis identified the edges with statistical significance (p < 0.01) at the group level, machine learning was able to select those features that were most important for individual classification – the increase/decrease ratio compared between the two approaches might not always and necessarily be consistent. Machine learning approaches are ideally suited to recover this granularity. Additionally, the L. Gamma results were mostly consistent with previously reported overall decreases in a range of metrics in the gamma frequency band observed from EEG, such as frontal nodal connection strength and communication efficiency^24^.

Our data-driven machine learning process was able to extract information that is in line with our knowledge of the neurobiology of PTSD. For example, Theta activity involving the right middle frontal gyrus were among the selected edges with decreased synchrony, including those synchronising with the left insula and right postcentral gyrus. For Alpha, edges involving the right superior parietal lobe and right supramarginal gyrus, left middle temporal gyrus and left middle temporal pole, as well as left precentral gyrus and left inferior frontal gyrus pars triangularis were among the top features differentiating PTSD and controls – other studies have previously reported dysfunction involving the superior parietal lobe, middle temporal gyrus, and left precentral gyrus^25^. Additionally, decreased synchrony between the amygdala and fusiform was found to be important for PTSD classification, in line with work from Stevens and colleagues where the weakened coupling between the amygdala and the prefrontal regions was also observed in PTSD conditions^26^. L. Gamma synchrony between the left hippocampus and right middle temporal pole also proved to be an important feature, consistent with our previous findings where MEG hyper-synchrony was observed at the group-level for PTSD^14^. Additionally, decreased synchrony between the thalamus and lingual gyrus across multiple frequencies might be related to previously reported findings reporting structural changes in these regions in PTSD^27^. Taken together, PTSD status was identified using our MEG-derived synchrony and our machine learning workflow, highlighting its potential as an application in this disorder and other neuropsychiatric disease, as well as a tool for hypothesis-generation and mechanistic exploration in MEG studies more generally.

Ultimately, a final SVM classification model was built using the feature-selected data. For the five frequency bands tested, the resulted final SVM models were significant (permutation p < 0.05) in classifying individuals with PTSD against the trauma-exposed controls according to the permutation tests using the training set. Using the independent test data set, the classification performance showed AUC values over 0.8 for all five frequency bands, suggesting excellent classification accuracy. Notably, Alpha and H. Gamma bands exhibited AUC value of 0.9. Such results were consistent with the previous studies where the Alpha and gamma activity were associated with PTSD in EEG^28,29^. Additionally, the classification capacity of the selected edges was independently tested using PLS-DA modelling. The results suggested that the high classification capability of the selected edges shown with the SVM modelling was also manifested in PLS-DA modelling, thus considered universal regardless of the classification method.

Notwithstanding the limitation of a small sample size in the context of artificial intelligence-based data mining, the present study leveraged machine learning for PTSD classification based off MEG-derived signatures. Further research with larger sample size including treatment outcome longitudinal studies is warranted. The machine learning analysis identified the minimal number of biologically relevant features that could serve as potential PTSD signatures. These functional connectivity biomarkers may be used for neuropathological research and classification modelling. The main contribution of the study was that we demonstrated the viability and promising results of the machine learning approach in identifying biological and functional relevant biomarkers from MEG functional connectome data for PTSD research, specifically in the context of combat-related cases. It should also be remembered that the PTSD group was tested against control participants who themselves were heavily traumatised with the same combat-related conditions, some with sub-threshold PTSD symptoms. As a secondary contribution, along with a machine learning framework, we provided a use case of the combination of machine learning and MEG functional connectome in biomarker discovery and classification modelling for neuropsychiatric disorders.

## Materials and methods

Details on the patient group demographics, data acquisition, and imaging analysis can be found in Dunkley *et al*.^14^. What follows below is a summary statement. Additional information regarding data collection, processing and machine learning analysis can be found in Supplementary Information S1. Data acquisition from the 2014 study^14^ as well as the current study were performed with the informed consent of each individual and under the approval of the Research Ethics Board at the Hospital for Sick Children (SickKids, http://www.sickkids.ca/Research/Research-Ethics/REB-office/index.html) in accordance to various ethics guidelines including Helsinki Declaration on Research Ethics.

### Participants

23 Canadian Armed Forces soldiers diagnosed with PTSD (all male, mean age = 37.4, SD = 6.8, age range 22–48) were recruited and had 5 minutes of eyes open MEG resting state data recorded. Twenty-one trauma-exposed peers (all male, mean age = 33.05, SD = 5.26, age range 18–45) who did not develop PTSD were recruited as a control group. The control subjects were deployed in the same battlefields as their peers and experienced similar traumatic events, including engagement of enemy combatants and threat to life.

All participants were recruited through the Canadian Armed Forces (CAF) Operational and Trauma Stress Support Centres (OTSSC). All participants were English-speaking and thus able to understand and answer to the instructions, questionnaires and consent. PTSD status was determined through a comprehensive semi-structured interview based on DSM-IV-T^5^ by a psychiatrist or psychologist specializing in trauma-related mental health injuries. CAF-standardized psychometric testing was also used during the process. More than one DSM-IV-TR “A1” related criteria were used to identify the traumatic event(s) contributing to PTSD development, namely “direct exposure, witnessing the trauma, learning that a relative or close friend was exposed to a trauma, indirect exposure to aversive details of the trauma, usually in the course of professional duties (e.g., first responders, medics)”.

For PTSD participants, comprehensive initial inclusion criteria were used, including combat-related PTSD diagnosis, presence of PTSD symptoms 1–4 years before the study, regular mental health follow-up, PCL score greater than 50 (severe PTSD), and no history of traumatic brain injuries. Additionally, as part of the inclusion process, the PTSD participants were interviewed by a psychiatrist, with their electronic health records screened. Moreover, the participants were subject to the Defence and Veteran’s Brain Injury Centre (DVBIC) three item screening tool. Furthermore, extensive exclusion conditions were also utilized for the participant recruitment, such as history of seizures or other neurological disorders and active substance abuse. Participants met conditions that would potentially impact MEG screening were also excluded, including presence of ferrous metal in the body, medical device implantation, as well as several ongoing medications (e.g. anticonvulsants, benzodiazepines, and/or GABA antagonists) that were proven to influence EEG screenings. Due to the nature of naturalistic samples, all PTSD participants were under treatment of one or more evidence-based psychotropic medications, example including selective serotonin reuptake inhibitors (SSRIs), serotonin-norepinephrine reuptake inhibitors (SNRIs) and Prazosin. Soldiers from the controls group were matched with the PTSD participants on ranks, education level, handedness and military experience.

### Magnetoencephalography

The details of MEG acquisition and analyses can be found in Dunkley *et al*.^14^; briefly, we acquired 151 channel MEG on a CTF system at the Hospital for Sick Children. MEG data were co-registered with an anatomical T1 MRI, and a beamformer was used to recover time series from 90 regions of the Automated Anatomical Labelling atlas (AAL)^30^. The weighted phase lag index (wPLI) was used to determine all pairwise combinations of seed synchrony^31^, with wPLI varying between 0 and 1, and used as the edge weight in the matrix. We tested canonical frequency ranges, included Theta (4–7 Hz), Alpha (8–14 Hz), Beta (15–30 Hz), Low Gamma (or L. Gamma, 30–80 Hz) and High Gamma (or H. Gamma, 80–150 Hz). Evaluating multiple frequency ranges allowed us to test the relative performance between the bands, predicting that those which showed the largest group differences previously would provide the greatest accuracy in delineating individual cases here.

### Machine learning downstream data analysis

A visual representation of the overall workflow for the downstream analysis can be viewed in Fig. 1. The entire downstream data analysis and visualization pipelines were carried out using our custom developed R packages via UNIX Bash scripting. Detailed description of each step in the workflow and software tools used can be found in Supplementary Information S1. The following core techniques are featured in the method: unsupervised clustering analyses with hierarchical clustering and principal component analysis (PCA), univariate statistical analysis, SVM-centric machine learning analysis, as well as partial least squares discriminant analysis (PLS-DA).

For machine learning analysis, the data was split by participants into training (80%) and test (20%) sets. All the feature selection, cross validation and final modelling steps were carried out only on the training set. The classification performance of the model was tested on the test set (Fig. 2). For training, we used 10-fold cross-validation (CV). The previously described recursive RF feature selection (rRF-FS) method^32^ (also refer to Supplementary Information S1 “*Recursive random forest feature selection*” section) was used for feature selection (FS). To avoid modelling bias from the FS process, rRF-FS was integrated into 10-fold CV, i.e. CV-SVM-rRF-FS. Therefore, the final feature selection result was a consensus list from the ten CV-SVM-rRF-FS iterations. Subsequently, data with the selected edges were used for the final SVM classification modelling step. Moreover, PLS-DA was conducted as an independent verification algorithm for the machine learning feature selection and modelling analysis.

A univariate analysis step was used to examine the group differences and identify the functional edges with statistically significant changes (p < 0.01) in connectivity. To evaluate the effectiveness of univariate analysis on identifying functional edges that reflect group difference, alpha was applied to the raw p-value to preserve maximum amount of data variance. Unsupervised clustering analyses were used at various points of the downstream analysis. Specifically, hierarchical clustering was used to explore the grouping pattern in synchrony between the participant groups, as well as between the edges. Instead of dimensionality reduction, PCA was used as an unsupervised clustering method to confirm the functional profile grouping for PTSD and control groups.

## Supplementary information

Supplementary information legends.

Supplementary information S1.

Supplementary information S2.

Supplementary table S1.

Supplementary table S2.

Supplementary figures.

## Data Availability

The full results and associated data can be viewed in supplementary information. Due to the patient consent policy, raw data access is considered on a case-by-case basis. Please contact the corresponding author if interested.
