# Supplementary material for: Classifying post-traumatic stress disorder using the magnetoencephalographic connectome and machine learning
Source: Sci Rep. 2020 Apr 3;10:5937. doi: 10.1038/s41598-020-62713-5 (PMC7125168; doi:10.1038/s41598-020-62713-5)
Supplement: Supplementary file 1 — Supplementary information legends. [file 41598_2020_62713_MOESM1_ESM.docx]

**Classifying post-traumatic stress disorder using the magnetoencephalographic connectome and machine learning**

Jing Zhang^1,2^, J. Don Richardson^3,4^ & Benjamin T. Dunkley^1,2,5^

^1^ Department of Diagnostic Imaging, Hospital for Sick Children, Toronto ON, Canada

^2^ Neurosciences & Mental Health, SickKids Research Institute, Toronto ON, Canada

^3^ St Joseph’s, London OSI, London ON, Canada

^4^ MacDonald Franklin OSI Research Centre, London ON, Canada

^5^ Department of Medical Imaging, University of Toronto, Toronto ON, Canada

**Corresponding Author:**

Jing Zhang

Office: 555 University Avenue, Toronto, M5G 1X8, Canada

Email: jzhangcad@gmail.com, jing.zhang@sickkids.ca

Research Fellow – Hospital for Sick Children

**Keywords:**

PTSD, machine learning, classification, neuronal oscillations, functional connectivity, resting-state, MEG

**Supplementary information legends**

**S1.** Supplementary methods

**S2.** Supplementary results

**Table S1.** Complete results for univariate analysis
**Table S2.** CV-SMV-rRF-FS results with univariate analysis stats.

**Fig. S1.** Heatmaps for hierarchical clustering analysis results using all edges for the five frequency bands. Dendrograms show the clusters for participants (columns) and the edges (rows). (A) Theta band, (B) Alpha band, (C) Beta band, (D) L. Gamma band, and (E) H. Gamma band.

**Fig. S2.** The volcano plots show the edges with significant changes (p < 0.01) in synchrony (red dots). Horizontal dashed line indicates the p value threshold (0.01) while the vertical line divides directionality (i.e. increases or decreases). (A) Theta band, (B) Alpha band, (C) Beta band, (D) L. Gamma band, and (E) H. Gamma band.

**Fig. S3**. Heatmaps for hierarchical clustering analysis results using only the significant edges (p < 0.01) for the Theta (A), Beta (B) and L. Gamma (C) bands. Z score is plotted for the heatmaps. dendrograms show the clusters for participants (columns) and the edges (rows).

**Fig. S4.** ROC-AUC results for the Theta (A), Beta (B) and (C) L. Gamma bands. AUC values are shown in the ROC plots.

**Fig. S5**. SVM model evaluation using permutation test. Permutation results showing the percentage accuracy of both the final SVM model and the permutation models, with dashed line indicating the final model accuracy level. Numbers on the x-axis are the models, with 0 representing the final SVM model. (A) Theta band, (B) Alpha band, (C) Beta band, (D) L. Gamma band, and (E) H. Gamma band.

**Fig. S6.** Score plots and biplots (i.e. score plot and loading plot) showing PCA result. For biplot, the loading plots exhibit the contribution of the edges to clustering pattern. Left column: Score plots for PCA results from all the edges; middle column: biplots for PCA results from data with only the significant edges (p < 0.01); right column: biplots for PCA results from data with CV-SVM-rRF-FS selected edges. (A) Theta band, (B) Beta band and (C) L. Gamma band.

**Fig. S7.**  Score plots and VIP plots showing PLS-DA results. Left column: PLS-DA score plots showing the supervised clustering pattern on both components upon PLS-DA modelling using the CV-SVM-rRF-FS selected edges; right column: PLS-DA VIP scores for both model components for all the CV-SVM-rRF-FS selected edges, with the horizontal dashed line indicating the importance threshold (0.8), and the codes on the x-axis representing the edges. (A) Theta band, (B) Beta band and (C) L. Gamma band.

**Fig. S8.** ROC-AUC and permutation for the PLS-DA models. Left column: ROC curve with AUC values for both components. Right column: Permutation test results showing RMSEP (root mean squared error of prediction) values for both final and permutation PLS-DA models for both participant groups; numbers on the x-axis are the models, with 0 representing the final SVM model. (A) Theta band, (B) Alpha band, (C) Beta band, (D) L. Gamma band, and (E) H. Gamma band.
