# Supplementary material for: Classifying post-traumatic stress disorder using the magnetoencephalographic connectome and machine learning
Source: Sci Rep. 2020 Apr 3;10:5937. doi: 10.1038/s41598-020-62713-5 (PMC7125168; doi:10.1038/s41598-020-62713-5)
Supplement: Supplementary file 2 — Supplementary information S1. [file 41598_2020_62713_MOESM2_ESM.docx]

**Classifying post-traumatic stress disorder using the magnetoencephalographic connectome and machine learning**

Jing Zhang^1,2^, J. Don Richardson^3,4^ & Benjamin T. Dunkley^1,2,5^

^1^ Department of Diagnostic Imaging, Hospital for Sick Children, Toronto ON, Canada

^2^ Neurosciences & Mental Health, SickKids Research Institute, Toronto ON, Canada

^3^ St Joseph’s, London OSI, London ON, Canada

^4^ MacDonald Franklin OSI Research Centre, London ON, Canada

^5^ Department of Medical Imaging, University of Toronto, Toronto ON, Canada

**Corresponding Author:**

Jing Zhang

Office: 555 University Avenue, Toronto, M5G 1X8, Canada

Email: jzhangcad@gmail.com, jing.zhang@sickkids.ca

Research Fellow – Hospital for Sick Children

**Keywords:**

PTSD, machine learning, classification, neuronal oscillations, functional connectivity, resting-state, MEG

**S1 - Supplementary methods**

*Unsupervised clustering analysis*

Hierarchical clustering and PCA were used as the unsupervised cluster methods at various points during the machine learning analysis. When carried out for all the unique edges, these steps explore the overall functional connectivity status for the PTSD and control groups when carried out. With feature-reduced data resulted from the subsequent univariate analysis and rRF-FS process, the unsupervised clustering analysis assesses grouping effectiveness for the statistically significant and machine learning selected edges, respectively. Firstly, hierarchical clustering analysis groups data both by participants and edges according to the data similarity. The clustering method “complete” was used for the analysis. As a visual representation, a hierarchical clustering heatmap with dendrograms were generated for each frequency band. Regarding PCA, the core principle of the analysis is to transform data into latent variables (LV, or principal component, PC in the context of PCA) in a linear and uncorrelated fashion. By decomposing and projecting data into a lower dimension space while retaining data variance, PCA can be used to identify the minimum number of the PCs that can explain maximal data variance (Lenz et al., 2016). As such, PCA enables a potentially viable routine to visualize the difference between the groups despite the high original dimensionality. Therefore, PCA presents an effective unsupervised clustering technique to assess data grouping pattern and complexity. PCA score plot are often used to show the clustering results. Moreover, a loading plot masked on the score plots (i.e. PCA biplot) is used to demonstrate the contribution of the data variables (i.e. features) to the clustering pattern. For the current study, the score plot was used for PCA with all edges, while biplot for the feature-reduced data by either univariate analysis or rRF-FS. Moreover, the first two PCs (with their respective percentage variance explained value) were used for the score plot or biplot. The R native clustering function and RBioFS package (Zhang et al., 2016) were used for the hierarchical clustering analysis and PCA, respectively. The hierarchical clustering heatmaps were generated with the R package gplots (Warnes et a., 2019).

*Univariate analysis*

The functional connectivity wPLI data was subject to a linear modelling and empirical Bayesian-based univariate analysis to assess the group difference. The core principle of the analysis was described previously by Smyth (2004) and Ritchie et al. (2015). In general, for each participant group, a linear model was constructed for each edge, modelling the raw values and the mean. A second linear model was then generated to model the changes between participant groups (i.e. fold change) given the contrast matrix and the mean wPLI value. The goal was to test the hypothesis of no change in wPLI between PTSD and control group. Given the parallel structure of the functional connectivity data featuring a large number of edges, it is beneficial to subsequently fit a hierarchical model for all the edges per sample group so that a robust statistical performance can be reached despite relatively small sample sizes. The estimation of the parameters of hierarchical model (i.e. hyperparameters) was achieved by the empirical Bayesian method where the hyperparameters are derived from the existing data. Albeit for a different purpose, empirical Bayesian method has previously been used as a normalization step during brain imaging data analysis (Chen et al., 2015). The current study thus extended the utility of the method to parametric hypothesis test for MEG functional connectivity data. We used the R package limma implementation of the method where the highly variable edges were treated separately during the empirical Bayesian-based hyperparameter estimation, ultimately resulting in an elevated statistical performance (Ritchie et al., 2015). Based on the core functions of the limma package, a custom R script was developed specifically compatible for our MEG functional connectivity data. It is worth noting that due to potential high level of similarity in the functional connectivity profile between the PTSD and trauma control groups was hypothesized even with a robust univariate statistical approach. Moreover, given serving as the initial group difference assessment method, as opposed to identifying edges for mechanistic exploration or biomarker discovery, a relatively liberal approach was used for statistical significance determination where the alpha (0.01) was applied to the raw p values.

*Support vector machine analysis*

An SVM-centric machine learning process was used to select most important edges and construct an optimal model for PTSD classification. In general, SVM classification relies on determining a separating hyperplane and utilization of kernel function (Boser et al., 1992). The separating hyperplane determination in SVM uses the maximum-margin principle where the hyperplane is selected with the maximum distance to the sample vectors. The sample vectors closest to the hyperplane are called support vectors. Moreover, SVM takes advantage of kernel functions to train classification model for the complex data that maximum-marge hyperplane would fail at separating data classes. Specifically, the kernel functions apply data transformation and added the transformed data back to the original dataset, thereby projecting the original data into higher dimensional space where the new maximum-margin hyperplane is able to classify the otherwise non-separable data. However, SVM is prone to overfitting, especially when using kernel function where new dimensions are added to the data vectors (Han and Jiang, 2014). As such, a feature selection process is often required for SVM modelling. Furthermore, while independent from the distribution of the training data, the SVM classifiers can only reach optimal performance when the test dataset shares the same distribution with the training set (Vapnik and Lerner, 1963). Therefore, several considerations were taken when implementing SVM for the current study. The current study incorporated an rRF-FS algorithm as the core feature selection strategy during SVM analysis (Zhang et al., 2016). Also, a k-fold nested-CV process was used for model selection. As opposed to placing feature selection before the SVM classification modelling process as a separate step, integrating the feature selection functionality into the nested CV step minimizes the bias generated from the feature selection algorithm, therefore avoiding model overfitting and bias (Cawley and Talbot, 2010). Additionally, a random resampling step was used to generate training and external test datasets, thereby providing unbiased SVM model evaluation while ensuring the aforementioned same data distribution condition.

A visualized workflow can be viewed in Fig. 2. First of all, data was partitioned by participants with 80% of the data used a the SVM training set and the rest 20% the external test set. The training set was then subject to a nested-CV process for feature selection and initial modelling performance evaluation, featuring a k-fold random resampling and thus an iterative CV-SVM-rRF-FS strategy. Specifically, the training data was randomly resampled into ten folds, with the nine folds used as the internal training set for feature selection and SVM modelling, and the remaining fold the internal test set. For each CV iteration, the internal training set underwent a rRF-FS step that selected the most important edges, based on which the dimensionally of the internal training set was reduced. Subsequently, the feature-reduced internal training set proceeded to SVM modelling, during which an inner ten-fold CV was carried out again for the SVM model hyperparameter grid search and CV SVM model evaluation. As such, each CV iteration produced a list of edges selected by rRF-FS and a CV SVM model accuracy value. As a result, ten edge lists were obtained upon the ten-fold nested CV process. A voting process was then carried out on the ten lists to generate the final consensus edge list. The CV SVM accuracy values were used as a reference for the hyperparameter estimation. A final SVM model was then built using the complete training set with only the consensus edges, during which another ten-fold CV procedure was carried out for the final hyperparameter optimization and internal model evaluation. A permutation test was used for model evaluation: the sample labels of the training data was permutated to generate a permutated SVM model with the same setups as the SVM modelling with the original training data (Ojala and Garriga, 2010). The permutation test was conducted 99 times to ensure a stable result. A hypothesis test was then carried out to test the difference in the prediction accuracy between the original and the permutation models. Additionally, a permutation p value was calculated according to Ojala and Garriga (2010). The SVM model with permutation p < 0.05 was considered significant in PTSD classification. Ultimately, the final SVM model was evaluated using the external test data. Receiver operating characteristic and area under curve (ROC-AUC) analysis was used as the SVM classification performance metric.

For the core SVM algorithm, we used the R implementation of the state-of-art libsvm library in the form of e1071 package (Meyer et al., 2019). The classification type was set to “C-classification” for a soft-margin method where outlier data points were taken into consideration during separating hyperplane determination. The radial basis function (RBF) was used as the kernel function for the current data. The rRF-FS component was achieved by the R package RBioFS (Zhang et al, 2016).

*Recursive random forest feature selection*

We incorporated an rRF-FS workflow in the SVM nested-CV framework for the feature selection step during SVM analysis. In general, RF is an evolution of the decision tree modelling (Breiman, 2001). The algorithm features bootstrap-based random feature bagging and iterative decision tree modelling. In addition to the conventional ROC-AUC and permutation-based model evaluation, RF model performance is also determined by the out-of-bag (OOB) error rate. Additionally, the RF-based modelling features a native variable importance assessment, via either permutation or gini score method (Nicodemus et al., 2010). Thanks to the random feature-bagging and bootstrap modelling, while not completely immune, the RF-based algorithms often demonstrate significant less risk for overfitting (Breiman, 2001; Segal, 2004). Taken together, we used RF as the core algorithm for feature selection, and our implementation had a heavy focus on stabilizing modelling performance via iteration and recursion. Specifically, a 50-time iteration was applied for all the RF modelling steps during rRF-FS.

As described previously (Zhang et al., 2016), the current rRF-FS process is a two-step FS framework. In the context of the current study, the univariate analysis-dependent -MEG functional connectivity data was first subject to an initial feature selection process. The initial selection step started with RF variable importance (VI) determination and ranking for the edges. The current study used mean decrease of accuracy as the VI value, which was calculated using a permutation test-based method. Specifically, to evaluate VI for a variable, the original RF model prediction accuracy was compared with the prediction accuracy of the RF model with permutated value for the said variable. Due to the iteration step, the edges were then ranked according to the mean VI value. To select the true important variables, a CART (classification and regression tree) modelling step was used where the edge ranking was modelled against the standard deviation of the VI values. The edges with VI value greater than one predicted minimum standard deviation from the CART model were selected. With only the edges selected from the initial selection step, the functional connectivity data was subject to a secondary selection step using a recursive sequential forward selection (SFS) algorithm. Briefly, edges selected from the initial selection were sequentially added to the RF modelling step according to the VI ranking. The OOB values of the sequential RF models were compared. The models reaching the minimum OOB plus one standard deviation were considered as the significant models, among which the one built with minimum number of edges was retained, and the said list of edges were considered the final selected edges. For RF modelling steps, we used 501 trees for decision tree generation.

*Partial least square discriminate analysis*

Generally, PLS-DA is a variation of PLS regression (PLSR) for classification problems (Lee et al., 2018). The core principle of the PLS algorithm is to generate a linear model between the responding variable(s) and the predictor variables upon LV projection (i.e. PLS components). As such, PLS shares similar transformation characteristics with PCA. The difference between the two methods is that PLS emphasizes the correlation between the responding variable(s) and the LVs, while PCA focuses on maximizing the variance reflected by the loading vector. Therefore, the unique property of PLS also positions the algorithm as a supervised clustering technique in addition to a classifier. Additionally, like RF, PLS provides a native variable importance in projection (VIP) score for assessing the contribution of the variables in the classification model, which is commonly used as a FS method for PLS modelling. The specific calculation was proposed by Eriksson et al (1999) and Tenenhaus (1998).

The present study uses PLS-DA as a companion analytic process to SVM modelling to (i) independently assess if the feature selection results from the CV-SVM-rRF-FS process were subject to method bias, and (ii) to evaluate the classification performance of the selected edges when using a different classification algorithm. Specifically, PLS-DA is conducted on the same training and test sets used in SVM analysis using the NIPALS (non-linear iterative partial least squares) algorithm (Wold et al., 2001). The first main task for PLS modelling optimization is selecting the optimal number of the LV feature space dimensions (i.e. PLS components) for building the classification model. The main goal is to identify the minimum number of PLS components that contain maximum correlation between the predictors and responding variable(s). Therefore, upon the initial PLS-DA modelling with all possible components, the model was subject to a component selection process in which the RMSEP (root mean square error of prediction) values were calculated for the components, and the first number of components reaching within one standard error of the minimum RMSEP were determined as optimal (Hastie et al., 2001). The optimal PLS-DA model was then built using the optimal number of components. To evaluate the performance of the model, RMSEP-based permutation test was conducted (999 iterations) (Ojala and Garriga, 2010). The PLS score plot was used to visualize the sample clustering pattern in the optimized component space in a supervised fashion. Additionally, ROC-AUC analysis was conducted using the test set. To independently assess the importance of the CV-SVM-rRF-FS selected edges, VIP was calculated. A bootstrap resampling step (50 iterations) was included in the calculation so that an accurate estimate could be achieved. Edges with a VIP value greater than 0.8 were considered important.

The PLS-DA process used in the present study was based on the R package pls implementation (Mevik et al., 2018) through custom-built R scripts. Additionally, data and results visualization was achieved through the R packages ggplot2 (Wickham, 2016).

**References**

American Psychiatric Association. Diagnostic and statistical manual of mental disorders 4th edn. (2013).

Boser, B. E., Guyon, I. M. & Vapnik, V. N. A training algorithm for optimal margin classifiers. in 5th Annual ACM Workshop on COLT. ed. Haussler, D. 144 – 152. Pittsburgh, PA: ACM Press. (1992).

Breiman, L. Random forests. *Mach Learn.* **4**, 5 – 32. (2001).

Cawley, G. C. & Talbot, N. L. On overfitting in model selection and subsequent selection in performance evaluation. *J Mach Learn Res.* **11**, 2079 – 2107. (2010).

Chen, S., Kang, J. & Wang, G. An empirical Bayes normalization method for connectivity metrics in resting state fMRI. *Front Neurosci.* **9**, 316. (2015).

Eriksson, L., Johansson, E., Kettaneh-Wold, N. & Wold, S. Introduction to multi- and megavariate data analysis using projection methods (PCA and PLS). Umea: Umetrics. (1999).

Han, H. & Jiang, X. Overcome support vector machine diagnosis overfitting. *Cancer Inform.* **13**, 145 – 58. (2014).

Hastie, T., Tibshirani, R. & Friedman, J. The Elements of Statistical Learning. New York, NY, USA: Springer New York Inc. (2001).

Lenz, M., Muller, F. J., Zenke, M. & Schuppert, A. Principal component analysis and the reported low intrinsic dimensionality of gene expression microarray data. *Sci Rep.* **6**, 25696. (2016).

Mevik, B-H., Wehrens, R. & Liland, K. H. pls: Partial Least Squares and Principal Component Regression. R package version 2.7-0. <https://CRAN.R-project.org/package=pls>. (2018).

Meyer, D., Dimitriadou, E., Hornik, K., Weingessel, A. & Leisch, F. e1071: Misc functions of the department of statistics, probability theory group (Formerly: E1071), TU Wien. R package version 1.7-1. <https://CRAN.R-project.org/package=e1071>. (2019).

Nicodemus, K. K., Malley, J. D., Strobl, C. & Ziegler, A. The behaviour of random forest permutation-based variable importance measures under predictor correlation. *BMC Bioinformatics.* **11**, 110. (2010).

Ojala, M. & Garriga, G. C. Permutation test for studying classifier performance. *J Mach Learn Res.* **11**, 1833 – 1863. (2010).

Ritchie, M. E. et al. limma powers differential expression analyses for RNA-sequencing and microarray studies. *Nuc Acids Res.* **43**, e47. (2015).

Segal, M. R. Machine learning benchmarks and random forest regression. Technical Report, Center for Bioinformatics & Molecular Biostatistics. San Francisco: University of California. (2004).

Smyth, G. K. Linear models and empirical Bayes methods for assessing differential expression in microarray experiments. *Statis appl genetics mol biol.* **3**(1), Article 3. (2004).

Tenenhaus, M. La régression PLS. Paris: Editions Technip. (1998).

Vapnik, V. & Lerner, A. Pattern recognition using generalized portrait method. *Autom Remote Control.* **24**, 774 – 780. (1963).

Warnes, G. R. et al. gplots: various R programming tools for plotting data. R package version 3.0.1.1. <https://CRAN.R-project.org/package=gplots>. (2019).

Wickham, H. ggplot2: Elegant graphics for data analysis. New York, NY, USA: Springer-Verlag New York. (2016).

Wold, S., Sjostrom, M. & Eriksson, L. PLS-regression: a basic tool of chemometrics. *Chemom Intell Lab Syst,* 58, 109 – 130. doi: 10.3390/s18051562. (2001).

Zhang, J., Hadj-Moussa, H. & Storey, K. B. Current progress of high-throughput microRNA differential expression analysis and random forest gene selection for model and non-model systems: an R implementation. *J Integr Bioinform.* **13**, 306. (2016).
