# Supplementary material for: Classifying post-traumatic stress disorder using the magnetoencephalographic connectome and machine learning
Source: Sci Rep. 2020 Apr 3;10:5937. doi: 10.1038/s41598-020-62713-5 (PMC7125168; doi:10.1038/s41598-020-62713-5)
Supplement: Supplementary file 3 — Supplementary information S2. [file 41598_2020_62713_MOESM3_ESM.docx]

**Classifying post-traumatic stress disorder using the magnetoencephalographic connectome and machine learning**

Jing Zhang^1,2^, J. Don Richardson^3,4^ & Benjamin T. Dunkley^1,2,5^

^1^ Department of Diagnostic Imaging, Hospital for Sick Children, Toronto ON, Canada

^2^ Neurosciences & Mental Health, SickKids Research Institute, Toronto ON, Canada

^3^ St Joseph’s, London OSI, London ON, Canada

^4^ MacDonald Franklin OSI Research Centre, London ON, Canada

^5^ Department of Medical Imaging, University of Toronto, Toronto ON, Canada

**Corresponding Author:**

Jing Zhang

Office: 555 University Avenue, Toronto, M5G 1X8, Canada

Email: jzhangcad@gmail.com, jing.zhang@sickkids.ca

Research Fellow – Hospital for Sick Children

**Keywords:**

PTSD, machine learning, classification, neuronal oscillations, functional connectivity, resting-state, MEG

**S2**

**Supplementary results**

*Hierarchical clustering analysis on all edges*

The results from the unsupervised clustering on all edges can be viewed in Fig. S1. As shown in the figure, in general, the functional connectivity profile clustering patterns failed to separate the control and PTSD groups for all five frequency bands. First, the results showed similar distribution of the wPLI data across all frequency bands tested. Moreover, frequency band-specific clustering status were observed. For example, Theta, Beta and L. Gamma bands clustering results exhibited two major clusters with relatively similar group sizes, whereas the Alpha and H. Gamma bands showed highly imbalanced clustering patterns. However, despite grouping into two clusters for all the frequency bands, none of the clustering pattern followed the PTSD and control grouping.

*Univariate analysis*

Following the current thresholding criteria, the linear modelling and empirical Bayesian-based univariate analysis identified edges with statistically significant changes in connectivity when comparing PTSD participants to the control group. The results were visualized with volcano plots showing significant edges and the directionality of the change (Fig. S2). Fig. A.2B shows the Theta band results. Specifically, 30 edges were identified as statistically significant in functional connectivity change between PTSD and control participants, with 15 edges showing increased connectivity. Regarding the Alpha band (Fig. S2B), the results suggested 40 edges with significant changes in the PTSD groups in relation to the control values, of which 22 increased in connectivity whereas 18 decreased. As seen Fig. S2C, Beta band included 30 significant increases and 19 decreases, making up a total of 49 significant edges. Fig. S2D indicates that 22 edges showed significant changes in functional connectivity between PTSD nd control groups in the L. Gamma band, with 7 increases and 15 decreases. In terms of the H. Gamma band (Fig. S2E), changes of 59 edges were considered as statistically significant between the PTSD and control groups, of which 26 were increases while 33 decreases.

*SVM modelling*

For the Theta band, the training set contained 21 PTSD and 15 control participants, with the rest used as test set, featuring 2 PTSD and 6 control participants. The nested CV step selected 11 edges and the accuracy at 0.62 ± 0.22 (mean ± SD). The selected edges included the ones involving right middle frontal gyrus. The final Theta band model generated with the selected edges included 13 support vectors and resulted in an internal CV accuracy at 0.94 ± 0.13. In terms of the Alpha band, the random partitioning resulted in 18 PTSD and 18 control participants for training set, while the rest 5 PTSD and 3 control participants were used as the test set. The rRF-FS step nested CV process identified 14 edges as the most relevant features for PTSD/control classification, such as the ones between right superior parietal lobe and right supramarginal gyrus, left middle temporal gyrus and left middle temporal pole, as well as left precentral gyrus and left inferior frontal gyrus pars triangularis. The nested CV accuracy for the Alpha band was 0.69 ± 0.21. The final Alpha band SVM model was then trained with 31 support vectors with an internal CV accuracy of 0.94 ± 0.12. Regarding the Beta band, equal number (i.e. 18) of PTSD and control participants were sampled as the training set, while the test set featured 5 and 3 participants from PTSD and control groups, respectively. The nested CV process selected 20 edges as the most important features, such as left amygdala: left fusiform gyrus. The nested CV accuracy was determined at 0.66 ± 0.26. As such, the subsequent final SVM model was built using 31 support vectors, with an internal CV accuracy of 0.97 ± 0.08. Moreover, the random partitioning for the L. Gamma band also yielded 18 PTSD and 18 control participants as the training set, with the 5 (PTSD) and 3 (control) participants used as the test set. The rRF-FS step during the nested CV process produced a list of 12 edges as the features optimal for PTSD/control classification, with edges between left hippocampus and right middle temporal pole included in the list. The nested CV accuracy for the L. Gamma band was determined at 0.8 ± 0.14. For the final L. Gamma SVM model, 18 support vectors were used; and the internal CV accuracy reached 0.97 ± 0.11. For the H. Gamma band, 20 PSTD and 16 control participants were randomly selected for the training set, while the function connectivity data from the rest 3 PTSD and 5 control participants served as the test set. Upon nested CV process, 19 edges were selected by the rRF-FS step as the most important features to differentiate the PTSD participants from the control group, such as edges involving left amygdala, left hippocampus and thalamus. The nested CV also led to a nested CV accuracy of 0.78 ± 0.2. For the final model, 36 support vectors were used, leading to an internal CV accuracy at 0.94 ± 0.11.

*Partial least squares discriminant analysis*

We used PLS-DA on the same training and test sets for SVM analysis to independently assess if the rRF-FS results were subject to method bias, as well as to evaluate the classification performance of nested CV-SVM-rRF-FS selected edges in the context another classification algorithm (Figs. 4, S7-S8). When used as a supervised clustering analysis, PLS-DA showed complete group separation across all the frequency bands using CV-SVM-rRF-FS selected data. Moreover, according to VIP analysis, most CV-SVM-rRF-FS selected edges were considered important by PLS-DA. It is worth noting that L. Gamma band exhibited the most edges below the VIP threshold (~ 2 edges) on both components (Fig. S7, right column). Permutation test using the training data set with 999 iterations showed that the lowest prediction error (i.e. RMSEP value) was found for the original model, leading to a p value of 0.001 across five frequency bands of interest (Fig. S8, right column). A summary of the PLS-DA results was included in Supplementary Material S2.

**PLS-DA summary**

*Theta*

PLS-DA machine learning for SVM results evaluation

=====================================================================

PLS-DA ncomp optimization

-------------------------------------

Optimal number of components: 2

PLS-DA permutation test

-------------------------------------

PLS-DA permutation results with 999 permutations:

comparison original.RMSEP p.value

1 ptsd 0.6381573 0.001

2 control 0.4904876 0.001

PLS-DA ROC-AUC

-------------------------------------

comp 1 AUC - control: 0.83

comp 1 AUC - ptsd: 0.83

comp 2 AUC - control: 0.83

comp 2 AUC - ptsd: 0.83

=====================================================================

*Alpha*

PLS-DA machine learning for SVM results evaluation

======================================================================

PLS-DA ncomp optimization

-------------------------------------

Optimal number of components: 2

PLS-DA permutation test

-------------------------------------

PLS-DA permutation results with 999 permutations:

comparison original.RMSEP p.value

1 ptsd 0.2661807 0.001

2 control 0.2661807 0.001

PLS-DA ROC-AUC

-------------------------------------

comp 1 AUC - control: 1

comp 1 AUC - ptsd: 1

comp 2 AUC - control: 1

comp 2 AUC - ptsd: 1

=====================================================================

Beta

PLS-DA machine learning for SVM results evaluation

=====================================================================

PLS-DA ncomp optimization

-------------------------------------

Optimal number of components: 2

PLS-DA permutation test

-------------------------------------

PLS-DA permutation results with 999 permutations:

comparison original.RMSEP p.value

1 control 0.2264985 0.001

2 ptsd 0.2264985 0.001

PLS-DA ROC-AUC

-------------------------------------

comp 1 AUC - ptsd: 0.93

comp 1 AUC - control: 0.93

comp 2 AUC - ptsd: 1

comp 2 AUC - control: 1

=====================================================================

Low gamma

PLS-DA machine learning for SVM results evaluation

======================================================================

PLS-DA ncomp optimization

-------------------------------------

Optimal number of components: 2

PLS-DA permutation test

-------------------------------------

PLS-DA permutation results with 999 permutations:

comparison original.RMSEP p.value

1 ptsd 0.2721295 0.001

2 control 0.2721295 0.001

PLS-DA ROC-AUC

-------------------------------------

comp 1 AUC - ptsd: 0.8

comp 1 AUC - control: 0.8

comp 2 AUC - ptsd: 0.87

comp 2 AUC - control: 0.87

=====================================================================

High gamma

PLS-DA machine learning for SVM results evaluation

======================================================================

PLS-DA ncomp optimization

-------------------------------------

Optimal number of components: 2

PLS-DA permutation test

-------------------------------------

PLS-DA permutation results with 999 permutations:

comparison original.RMSEP p.value

1 ptsd 0.6024932 0.001

2 control 0.5018834 0.001

PLS-DA ROC-AUC

-------------------------------------

comp 1 AUC - ptsd: 0.87

comp 1 AUC - control: 0.87

comp 2 AUC - ptsd: 0.93

comp 2 AUC - control: 0.93

=====================================================================
