# Supplementary material for: Classifying post-traumatic stress disorder using the magnetoencephalographic connectome and machine learning
Source: Sci Rep. 2020 Apr 3;10:5937. doi: 10.1038/s41598-020-62713-5 (PMC7125168; doi:10.1038/s41598-020-62713-5)
Supplement: Supplementary file 6 — Supplementary figures. [file 41598_2020_62713_MOESM6_ESM.pdf]

Figure S1

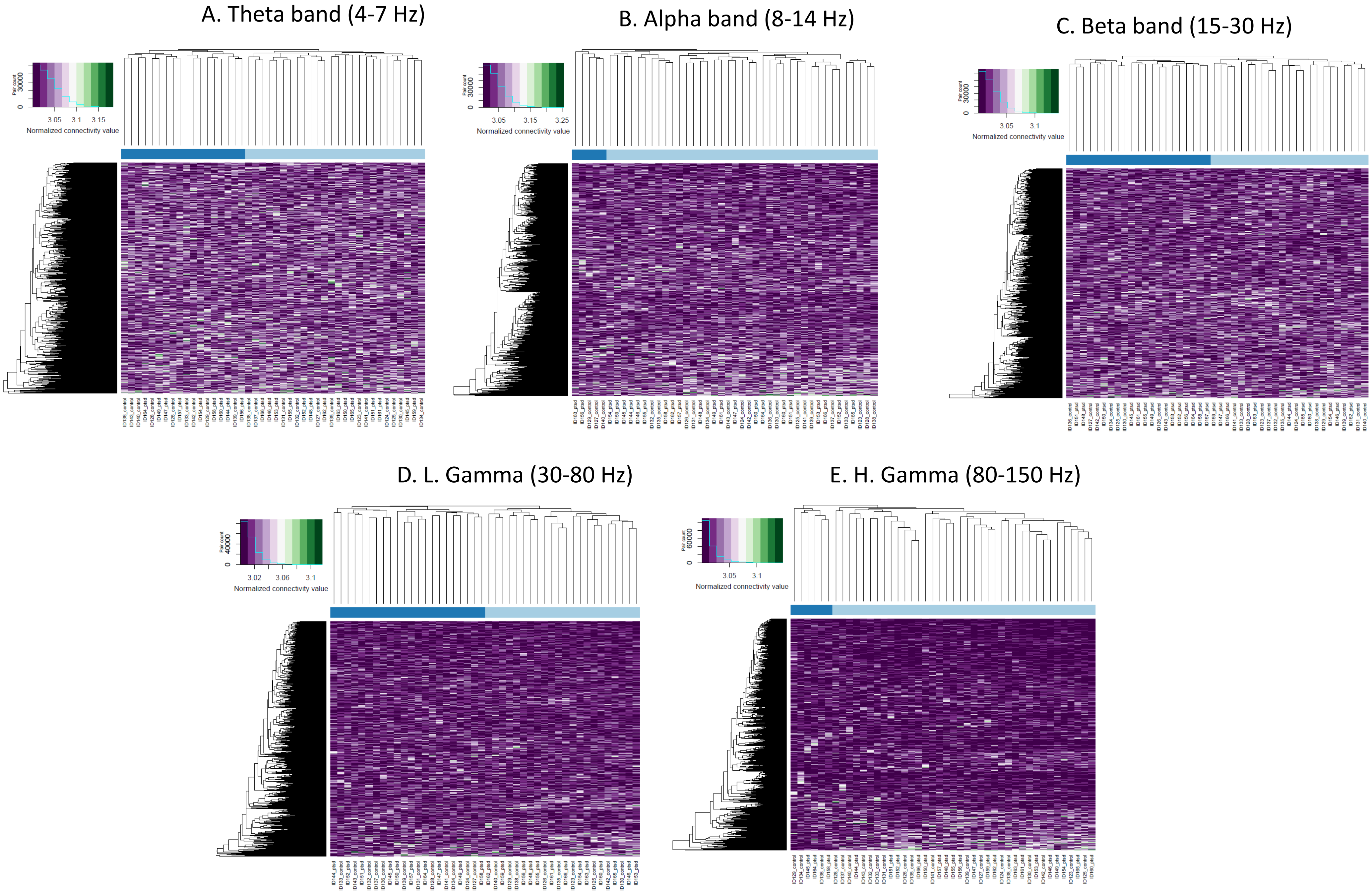
A. Theta band (4-7 Hz)
B. Alpha band (8-14 Hz)
C. Beta band (15-30 Hz)
D. L. Gamma (30-80 Hz)
E. H. Gamma (80-150 Hz)
Normalized connectivity value
Pair count

Figure S2

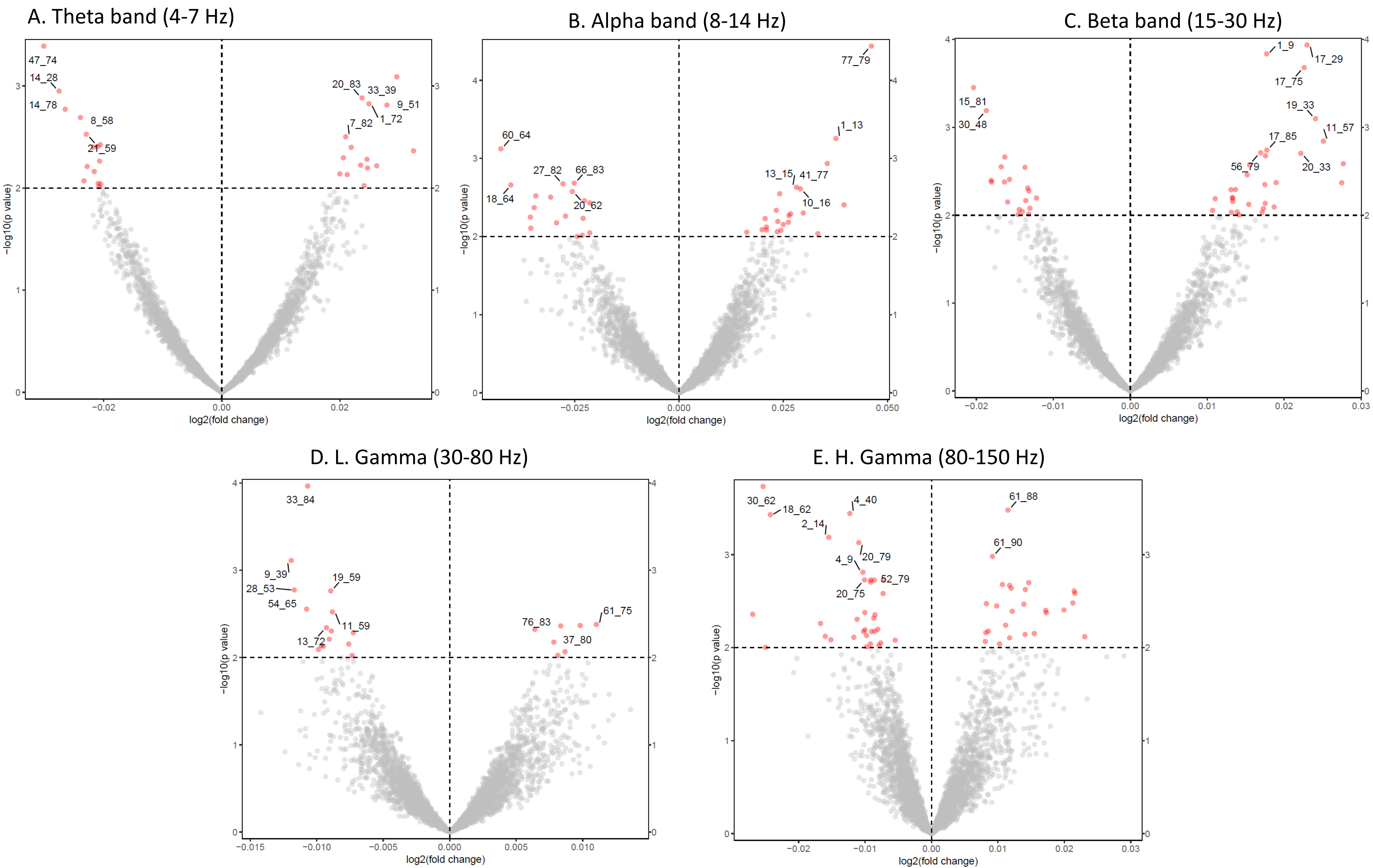

A. Theta band (4-7 Hz)
B. Alpha band (8-14 Hz)
C. Beta band (15-30 Hz)
D. L. Gamma (30-80 Hz)
E. H. Gamma (80-150 Hz)
-log10(p value)
log2(fold change)
47_74
14_28
14_78
8_58
21_59
20_83
33_39
9_51
1_72
7_82
77_79
60_64
1_13
27_82
66_83
18_64
20_62
13_15
41_77
10_16
1_9
17_29
17_75
15_81
30_48
19_33
11_57
17_85
56_79
20_33
33_84
9_39
28_53
54_65
19_59
11_59
13_72
76_83
61_75
37_80
30_62
18_62
2_14
4_40
4_9
20_79
52_79
20_75
61_88
61_90

Figure S3

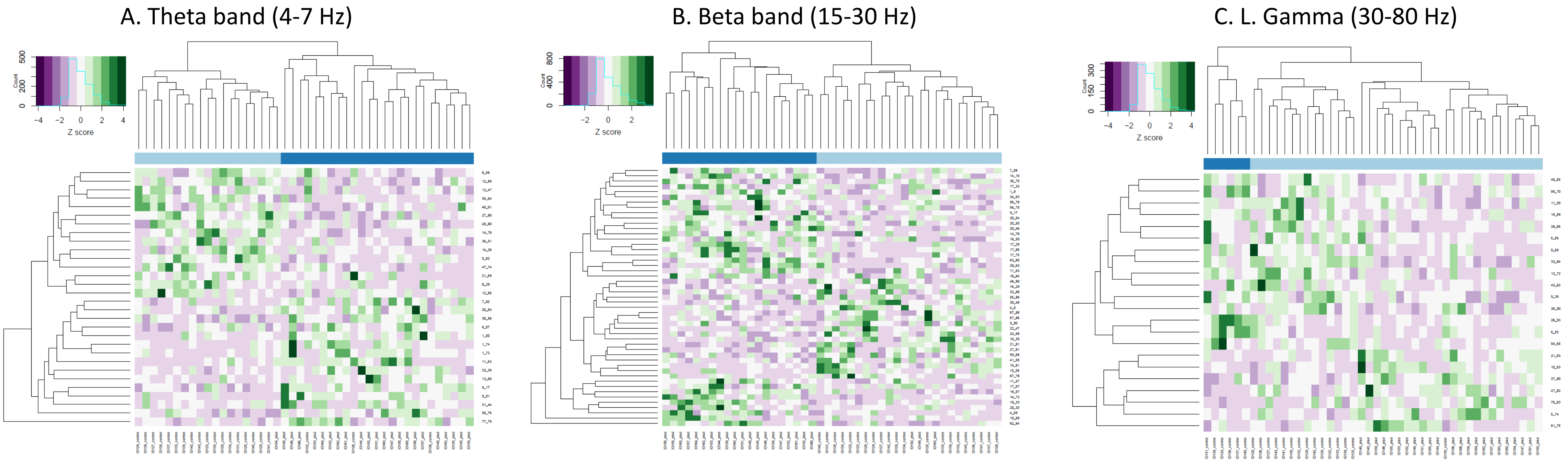
A. Theta band (4-7 Hz)
B. Beta band (15-30 Hz)
C. L. Gamma (30-80 Hz)
Count
Z score

Figure S4

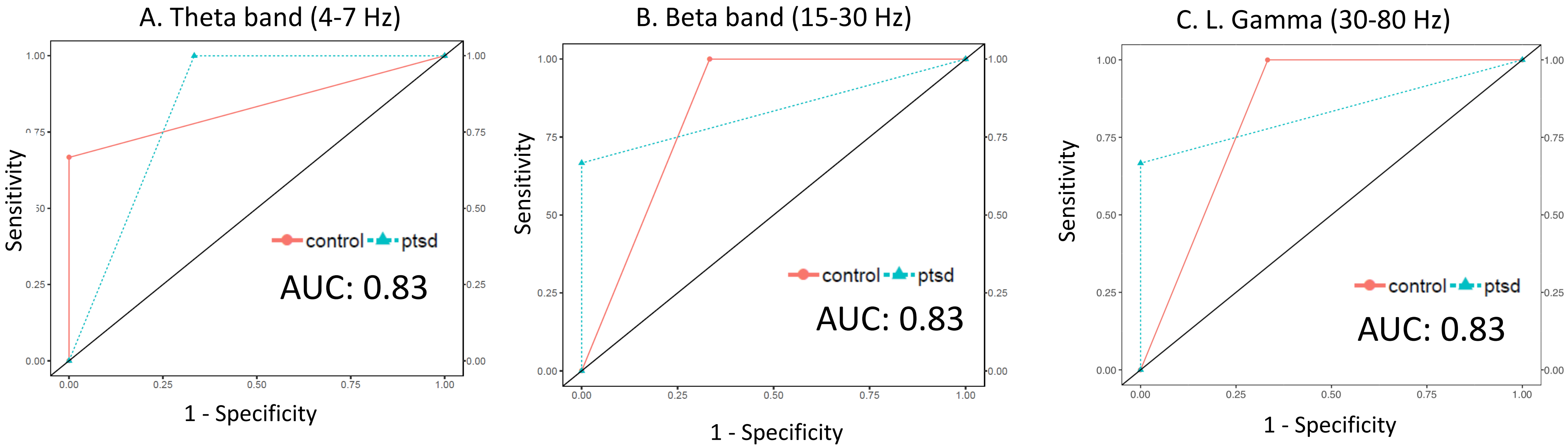

A. Theta band (4-7 Hz)
B. Beta band (15-30 Hz)
C. L. Gamma (30-80 Hz)
Sensitivity
1 - Specificity
control
ptsd
AUC: 0.83
AUC: 0.83
AUC: 0.83

Figure S5

A. Theta band (4-7 Hz)

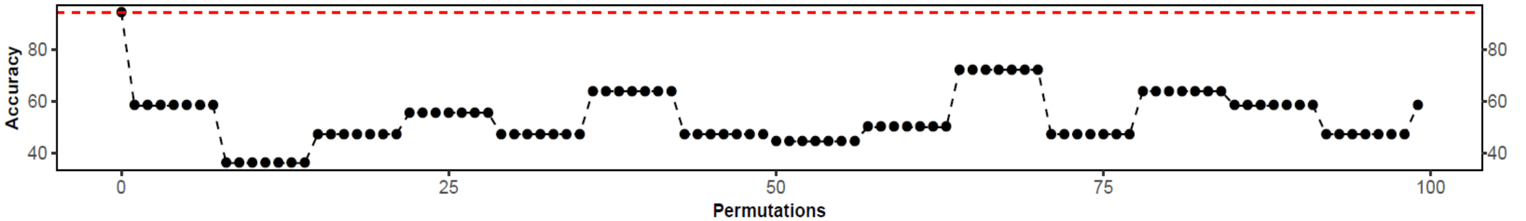

B. Alpha band (8-14 Hz)

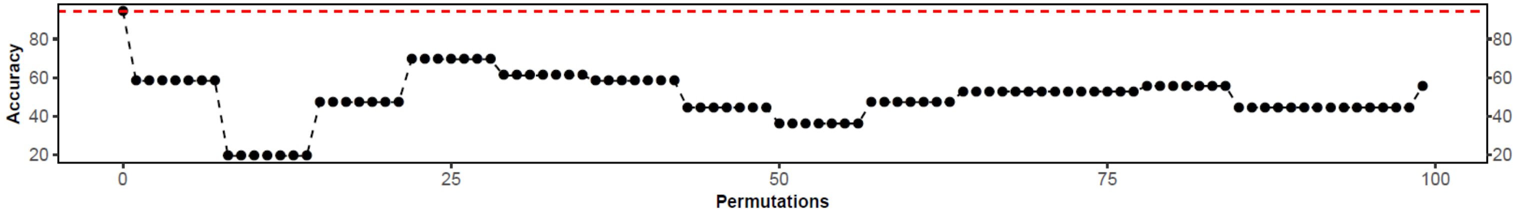

C. Beta band (15-30 Hz)

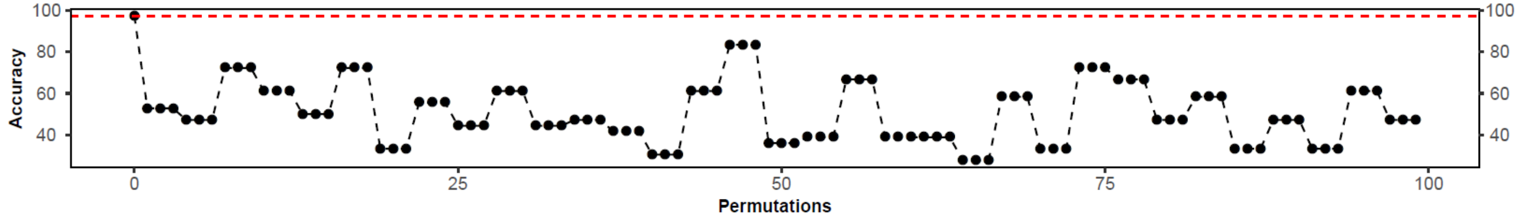

D. L. Gamma (30-80 Hz)

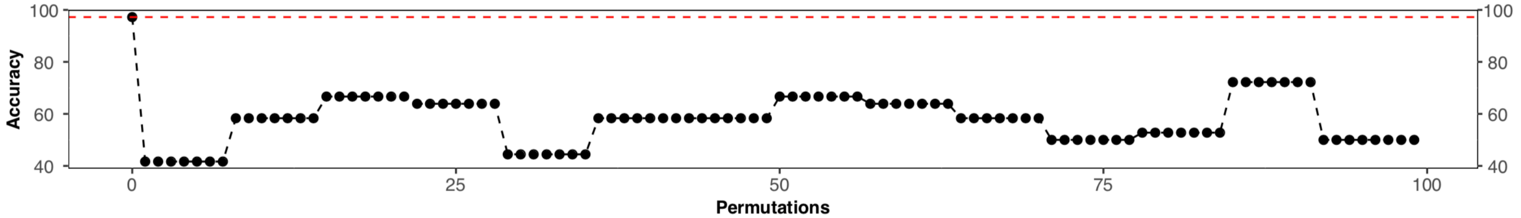

E. H. Gamma (80-150 Hz)

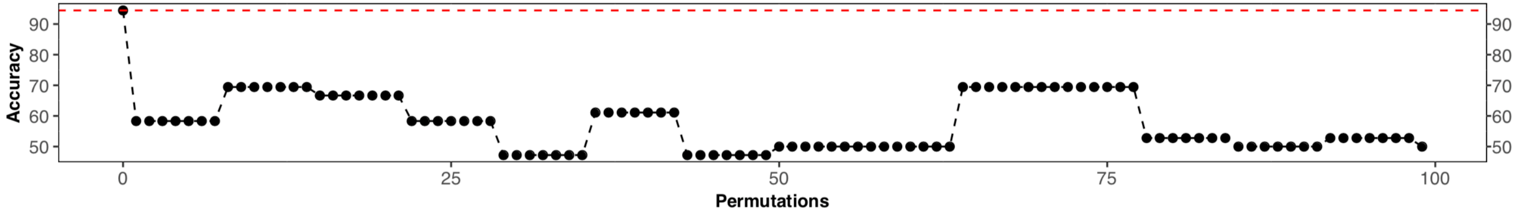

Figure S6

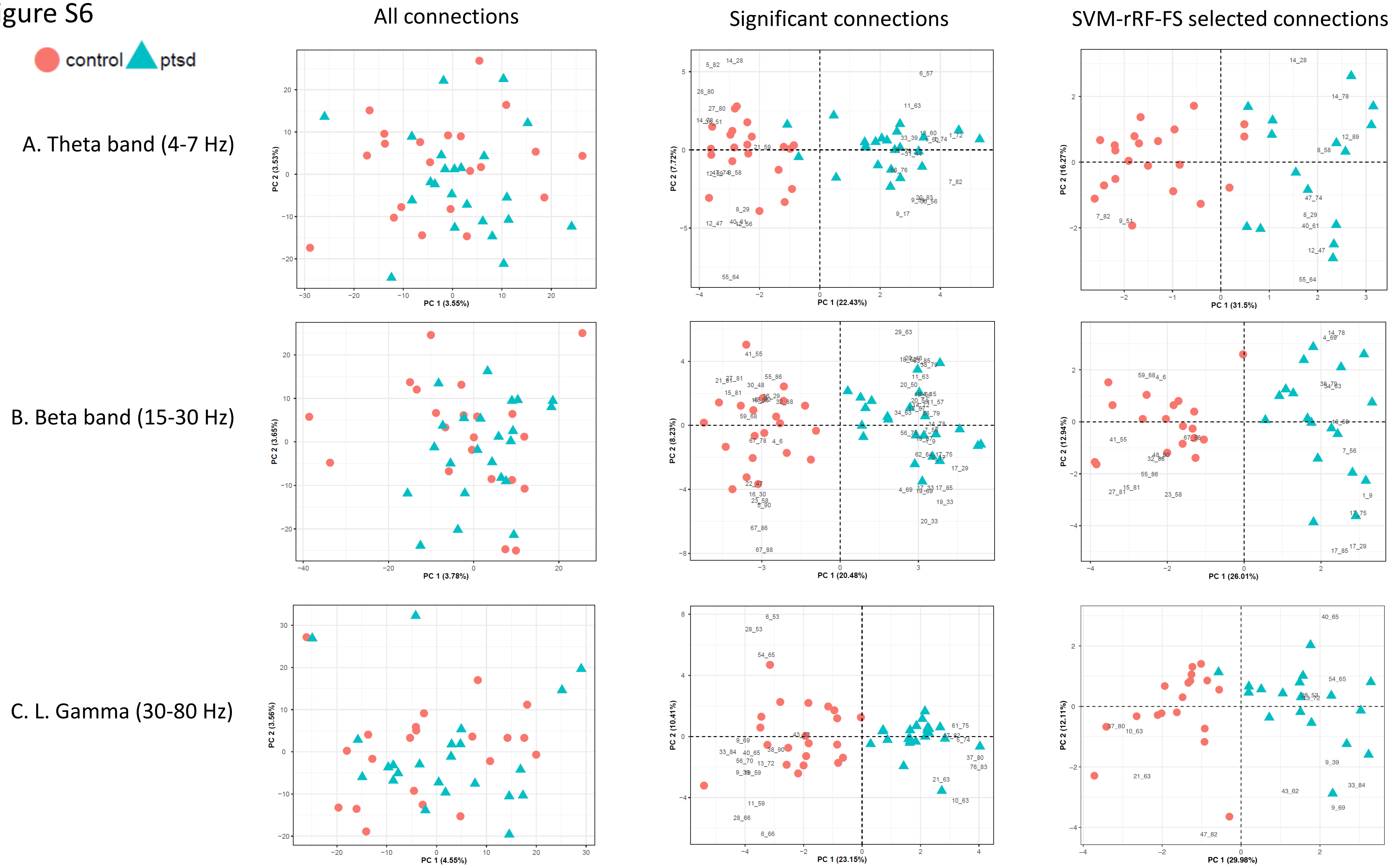
control
ptsd
All connections
Significant connections
SVM-rRF-FS selected connections
A. Theta band (4-7 Hz)
B. Beta band (15-30 Hz)
C. L. Gamma (30-80 Hz)
PC 1 (3.55%)
PC 2 (3.53%)
PC 1 (22.43%)
PC 2 (7.72%)
PC 1 (31.5%)
PC 2 (16.27%)
PC 1 (3.78%)
PC 2 (3.65%)
PC 1 (20.48%)
PC 2 (8.23%)
PC 1 (26.01%)
PC 2 (12.94%)
PC 1 (4.55%)
PC 2 (3.56%)
PC 1 (23.15%)
PC 2 (10.41%)
PC 1 (29.98%)
PC 2 (12.11%)

Figure S7

Score plot

PLS-DA variable importance in projection

A. Theta band (4-7 Hz)

B. Beta band (15-30 Hz)

C. L. Gamma (30-80 Hz)

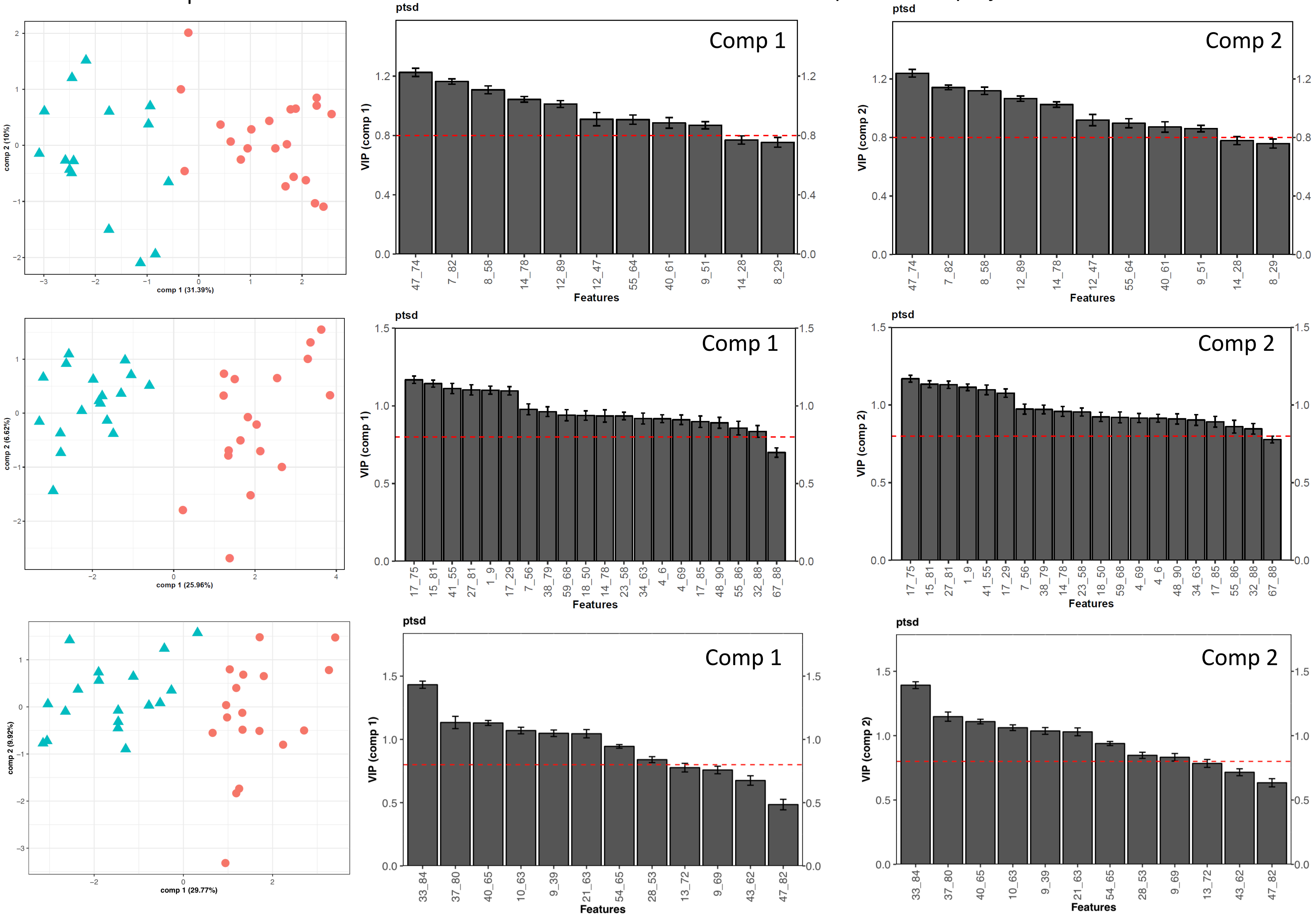

Figure S8-1

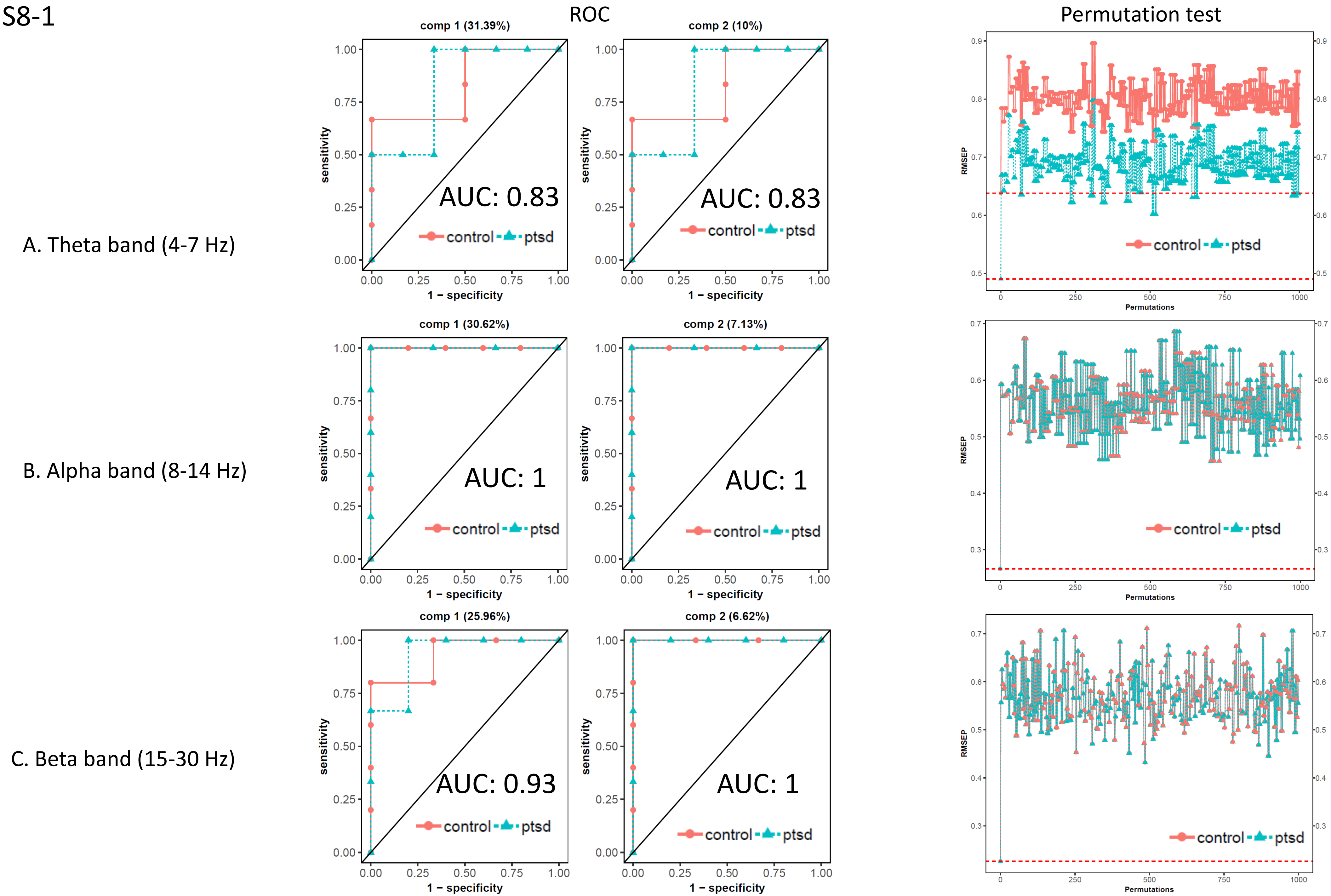

ROC
Permutation test
A. Theta band (4-7 Hz)
comp 1 (31.39%)
AUC: 0.83
comp 2 (10%)
AUC: 0.83
B. Alpha band (8-14 Hz)
comp 1 (30.62%)
AUC: 1
comp 2 (7.13%)
AUC: 1
C. Beta band (15-30 Hz)
comp 1 (25.96%)
AUC: 0.93
comp 2 (6.62%)
AUC: 1
sensitivity
1 – specificity
RMSEP
Permutations
control
ptsd

Figure S8-2

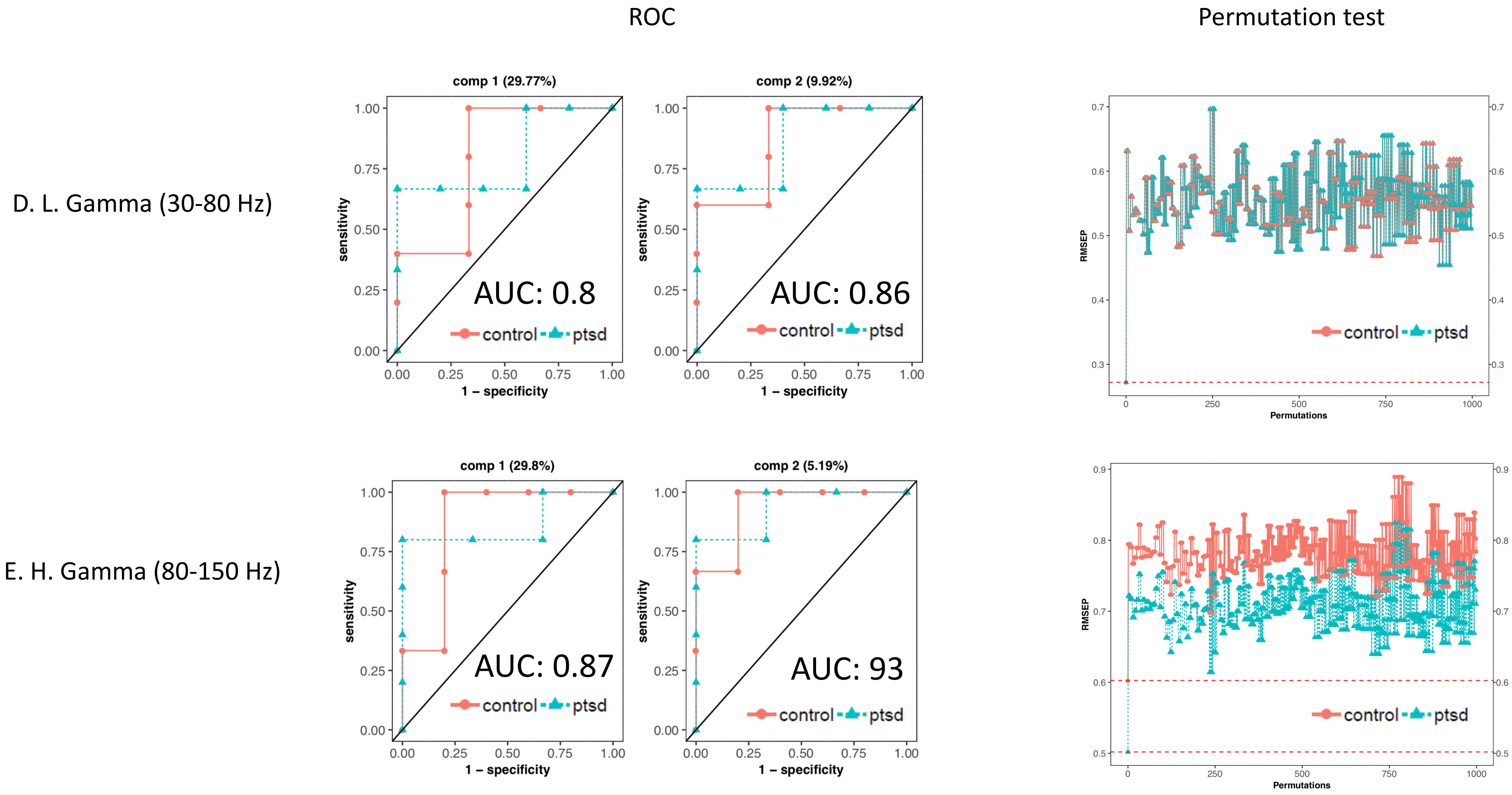
ROC
Permutation test
D. L. Gamma (30-80 Hz)
comp 1 (29.77%)
AUC: 0.8
comp 2 (9.92%)
AUC: 0.86
E. H. Gamma (80-150 Hz)
comp 1 (29.8%)
AUC: 0.87
comp 2 (5.19%)
AUC: 93
sensitivity
1 – specificity
0.00
0.25
0.50
0.75
1.00
control
ptsd
RMSEP
Permutations
0
250
500
750
1000
0.3
0.4
0.5
0.6
0.7
0.8
0.9
